# Screening of key functional components of Taohong Siwu Decoction on ischemic stroke treatment based on multiobjective optimization approach and experimental validation

**DOI:** 10.1186/s12906-023-03990-1

**Published:** 2023-06-01

**Authors:** Anqi Xu, Wenxing Li, Jieqi Cai, Zhuohua Wen, Kexin Wang, Yupeng Chen, Xifeng Li, Daogang Guan, Chuanzhi Duan

**Affiliations:** 1 Department of Cerebrovascular Surgery, Neurosurgery Center, Zhujiang Hospital, Southern Medical University, No.253. Gongye Middle Avenue, Haizhu District, Guangzhou, 510280 Guangdong China; 2 Department of Biochemistry and Molecular Biology, School of Basic Medical Sciences, Southern Medical University, Guangzhou, 510515 Guangdong China; 3 Guangdong Provincial Key Laboratory of Single Cell Technology and Application, Southern Medical University, Guangzhou, 510515 Guangdong China

**Keywords:** Ischemic stroke, Traditional chinese medicine, Network analysis, Multiobjective optimization, Oxygen-glucose deprivation and reoxygenation

## Abstract

**Background:**

Taohong Siwu Decoction (THSWD) is a widely used traditional Chinese medicine (TCM) prescription in the treatment of ischemic stroke. There are thousands of chemical components in THSWD. However, the key functional components are still poorly understood. This study aimed to construct a mathematical model for screening of active ingredients in TCM prescriptions and apply it to THSWD on ischemic stroke.

**Methods:**

Botanical drugs and compounds in THSWD were acquired from multiple public TCM databases. All compounds were initially screened by ADMET properties. SEA, HitPick, and Swiss Target Prediction were used for target prediction of the filtered compounds. Ischemic stroke pathological genes were acquired from the DisGeNet database. The compound–target–pathogenic gene (C-T-P) network of THSWD was constructed and then optimized using the multiobjective optimization (MOO) algorithm. We calculated the cumulative target coverage score of each compound and screened the top compounds with 90% coverage. Finally, verification of the neuroprotective effect of these compounds was performed with the oxygen-glucose deprivation and reoxygenation (OGD/R) model.

**Results:**

The optimized C-T-P network contains 167 compounds, 1,467 predicted targets, and 1,758 stroke pathological genes. And the MOO model showed better optimization performance than the degree model, closeness model, and betweenness model. Then, we calculated the cumulative target coverage score of the above compounds, and the cumulative effect of 39 compounds on pathogenic genes reached 90% of all compounds. Furthermore, the experimental results showed that decanoic acid, butylphthalide, chrysophanol, and sinapic acid significantly increased cell viability. Finally, the docking results showed the binding modes of these four compounds and their target proteins.

**Conclusion:**

This study provides a methodological reference for the screening of potential therapeutic compounds of TCM. In addition, decanoic acid and sinapic acid screened from THSWD were found having potential neuroprotective effects first and verified with cell experiments, however, further in vitro and in vivo studies are needed to explore the precise mechanisms involved.

**Supplementary Information:**

The online version contains supplementary material available at 10.1186/s12906-023-03990-1.

## Introduction

Stroke is the second leading cause of death in the world, and ischemic stroke accounts for approximately 80% of the total stroke incidence [1]. Ischemic stroke is a serious cerebrovascular event, in which cerebral blood flow is interrupted by cerebral vascular occlusion or rupture. It can lead to various nerve injuries and may be accompanied by serious complications [2]. For acute ischemic stroke, alteplase can be used for thrombolysis in time, and anti-small platelet drugs such as aspirin and clopidogrel can be used to increase blood flow [3]. However, even if blood perfusion in the cerebral infarction area is restored in time, complex pathological factors such as excessive inflammation, oxygen free radicals, neuronal excitotoxicity, and blood-brain barrier damage will still cause severe neuronal damage [4].

Ischemic stroke has complex pathological factors, however, currently, there is still lack of neuroprotective drugs. Small molecular drugs commonly used in clinic mainly include edaravone which can promote the scavenging of oxygen free radicals, citicoline which can stabilize cell membrane, and nimodipine which can antagonize calcium channels. Because their current effect on poststroke damage is not satisfactory, the development of novel neuroprotective drugs is still of great significance. TCM has a long history of treating stroke, and contains rich clinical experience, which provides a new exploration direction for exploring stroke drugs [5]. Currently, some TCM preparations, herbal extracts, and isolated potential active compounds have been confirmed in clinical trials, and are widely used in clinical treatment of stroke. For example, modern TCM injections such as Xingnaojing Injection, Naoxintong capsule, and Shenfu injection are reprocessed from TCM formulas Angong Niuhuang pill, Xiaoxuming Decoction, and Shenfu Decoction. In addition, some effective compounds, including Salvia miltiorrhiza polyphenolic acid, notoginseng saponin, hirudin, butylphthalide, and so on, are extracted from Salvia miltiorrhiza, notoginseng, leech, celery, and other herbs. These injections and compounds have been used for many years in Chinese stroke patients with good results [5–7].

At present, for decoding the underlying mechanism of TCM prescriptions on complex diseases, a systematic pharmacological analysis process has been established, which combines the collection of all components of botanical drugs, active component screening, target prediction, pathway analysis, and mechanism exploration [8, 9]. This process has successfully helped analyze the key functional compounds and mechanisms of many prescriptions used in complex diseases [10, 11]. For some examples, Wang figured out the key components of Chai-Hu-Shu-Gan-San and decoded the mechanism of treating depression by regulating downstream genes through protein kinase A or C to treat depression after cascade signal changes [12]; Chen found critical ingredients and mechanisms of Xuebijing injection in treating sepsis synergistically by affecting genes such as TAK1, TNF-α, IL-1β, and MEK1 in the MAPK, NF-κB, PI3K-AKT, Toll-like receptor, and TNF signaling pathways [13].

In the TCM treatment of complex diseases such as ischemic stroke, a complex network composed of multiple components and corresponding multiple targets has been formed [14]. This network usually contains thousands of compounds, which may be functional, ancillary, useless, or toxic [15]. How to obtain the key functional components related to diseases more accurately and conveniently is one of the important goals of network analysis of TCM. However, the traditional network analysis method mainly determines the key nodes according to the degree of connectivity in the network. Unfortunately, this does not correspond well to the complex pathological genes of stroke, and ignores the one-way propagation coefficient in the network from drug targets to pathological genes [16]. Therefore, the choice of the key functional components is often missing and incomplete. We hope to design a novel strategy combined with MOO model for analyzing stroke transcriptome to solve this problem.

In this study, we used THSWD as an example and used this model to screen key functional components in the treatment of ischemic stroke with the MOO model. THSWD comes from the “Golden Mirror of Medicine” and is now also included in the “Catalogue of Ancient Classic Famous Prescriptions”. THSWD comprises 6 herbs, each 15 g: *Paeonia obovate* (Chi Shao), *Cnidium Officinale* (Chuan Xiong), *Angelica sinensis* (Dang Gui), *Radix Rehmannia* (Di Huang), *Carthamus tinctorius* (Hong Hua), and *Prunus persica* (Tao Ren) (Table 1) [17]. Previous pharmacological studies have indicated that THSWD exhibited certain neuroprotective effects and potential therapy in vivo and *in vitro.* For example, Yun Shi found that a concentration of drug-containing serum of THSWD protected PC12 cells against OGD/R injury by heightening mitophagy and suppressing the activation of NLRP3 inflammasome [18]; Ni Wang verified that THSWD regulated Cell necrosis and neuroinflammation in the rat middle cerebral artery occlusion (MCAO) model [19]; Mengmeng Wang also reported that THSWD could reduce inhibit pyroptosis in MCAO rats [20]. Furthermore, some active ingredients of THSWD in stroke treatment have been reported sporadically. For example, ligustilide, a main lipophilic component isolated from *Cnidium officinale* and *Angelica sinensis*, was reported to attenuate ischemia reperfusion-induced neuronal apoptosis via multiple signaling pathways, including PI3K/AKT, MAPK, and caspase3 [21–23]. Hydroxy safflor yellow A (HSYA), as a major ingredient of *Carthamus tinctorius*, was reported to protect neurons after stroke by blocking HIF-1α/NOX2 signaling cascades to participate in antioxidative activity [24, 25]. Although the THSWD and several components in this prescription were reported with the potential therapeutic effect of ischemic stroke, the key functional components and the underlying mechanism of action are still poorly understood. Revealing the material basis and molecular mechanism of THSWD in the treatment of ischemic stroke with the MOO approach and experimental validation was suitable and necessary.

**Table 1:** The component, origin, and dosage of botanical drugs in THSWD.

Chinese name	Latin name	Native range	Dose
Bai Shao	*Paeonia lactiflora* Pall. (Paeoniaceae, Paeoniae Radix Alba)	Anhui, Sicuan, Zhejiang	15 g
Chuan Xiong	*Ligusticum chuanxiong* Hort. (Umbelliferae, Chuanxiong Rhizoma)	Sicuan, Jiangxi, Hunan, Hubei	15 g
Dang Gui	*Angelica sinensis* (Oliv.) Diels (Apiaceae, Angelicae Sinensis Radix)	Gansu, Yunnan, Qinghai	15 g
Di Huang	*Rehmannia glutinosa* Libosch. (Orobanchaceae, Rehmanniae Radix)	Shandong, Shanxi, Henan, Hebei	15 g
Hong Hua	*Carthamus tinctorius* L. (Asteraceae, Carthami Flos)	Henan, Hubei, Sicuan, Yunnan, Zhejiang	15 g
Tao Ren	*Prunus persica* (L.) Batsch (Rosaceae, Persicae Semen)	Beijing, Hebei, Shanxi	15 g

**Fig. 1:**
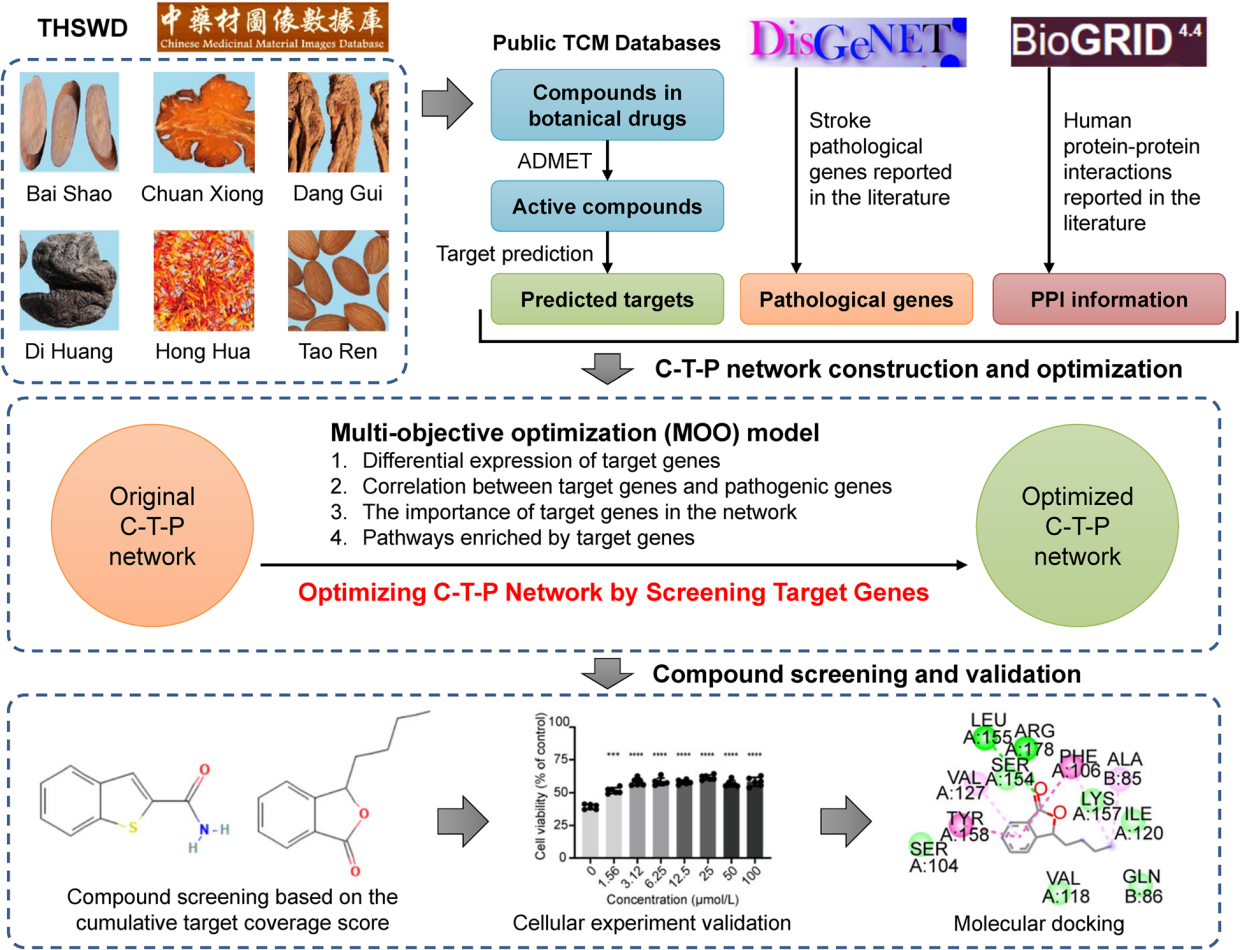
The flowchart of this study. Compounds of THSWD were acquired from public TCM databases. Target prediction was performed for ADMET-filtered compounds. The potential active compound-predicted targets and stroke pathological genes were used to construct the compound–target–pathological gene (C-T-P) network, followed by network optimization. The final compounds were screened based on the cumulative target coverage score. Cellular experiments and molecular docking were performed on the final screened compounds

The workflow of this study is shown in Fig. 1. First, a C-T-P network was constructed using chemical constituents collected from botanical drugs contained in THSWD. Then, the MOO model was used to optimize the network. Last, the key functional ingredients in THSWD were screened with cumulative target coverage scores and chosen to conduct experimental verification. The MOO model was designed to consider the differential expression of targets, the correlation between targets and pathogenic genes, the importance of targets in the C-T-P network, and the pathways enriched by targets and could be used to optimize the network from multiple perspectives to screen the key functional compounds. Furthermore, our study could also provide a theoretical basis and a methodological reference for the screening of natural small molecule compounds of Chinese herbal medicine.

## Materials and methods

### Herbs and compounds of THSWD

Botanical drugs and dosage information on THSWD were acquired from the Chinese Pharmacopeia 2020 (National Pharmacopoeia Commission, 2020). All botanical drugs were validated taxonomically by the Plants of the World Online (https://powo.science.kew.org/) and Chinese Medicinal Material Images Database (https://library.hkbu.edu.hk/electronic/libdbs/mmd/) (Table 1). Information concerning the chemical components in these botanical drugs was obtained from multiple public TCM databases, including the Traditional Chinese Medicine Systematic Pharmacology (TCMSP, https://tcmsp-e.com/) database [26], the Traditional Chinese Medicine Information Database (TCMID, http://bidd.group/TCMID/) [27], and the Traditional Chinese Medicine and Active Ingredient Database (TCMAID, http://bidd.group/TCMID/). Chemical structures were prepared and converted into canonical SMILES using the OpenBabel Toolkit [28].

### Screening of potentially active compounds in THSWD

The ADMET properties and physicochemical properties of all compounds were predicted using the ADMETlab 2.0 web server (https://admetmesh.scbdd.com/) [29]. According to Lipinski’s rule of five (Pollastri, 2010), potential active compounds met the following conditions: (1) molecular weight (MW) ≤ 500; (2) number of hydrogen bond donors (nHD) ≤ 5; (3) number of hydrogen bond acceptors (nHA) ≤ 10; (4) logarithm of the n-octanol/water distribution coefficient (logP) ≤ 5; and (5) number of rotatable bonds (nRot) ≤ 10. Furthermore, potential active compounds should also meet the following additional conditions: (6) human oral bioavailability 20% (F(20%)) ≤ 0.7; (7) blood‒brain barrier (BBB) penetration ≤ 0.7; (8) human ether-a-go-go related gene (hERG) blockers ≤ 0.7; (9) human hepatotoxicity (H-HT) ≤ 0.7; and (10) topological polar surface area (TPSA) ≤ 140.

### Compound-target prediction

The potential targets of the potential active compounds were predicted by using the following web servers: similarity ensemble approach (SEA) search server (https://sea.bkslab.org/) [30], HitPickV2 (http://www.hitpickv2.com/) [31], and SwissTargetPrediction (http://www.swisstargetprediction.ch/index.php) [32]. The union set of the prediction results is defined as the potential targets.

### Collection of ischemic stroke pathological genes

Stroke pathological genes were searched and downloaded from the DisGeNet database (https://www.disgenet.org/) [33]. We searched all ischemic stroke-related diseases and their corresponding genes. Genes with literature reports were defined as stroke pathological genes. The intersection of targets and pathogenic genes was defined as essential common proteins (ECPs).

### C-T-P network construction

All protein-protein interaction information was downloaded from the BioGrid database (https://thebiogrid.org/) [34]. We retrieved the interactions between targets and pathogenic genes and extracted protein‒protein interactions that were reported in the literature. Combining the previous compound–target prediction results, the C-T-P network was constructed and visualized with Gephi software (https://gephi.org/).

### Ischemic stroke transcriptome data analysis

Transcriptome data from the cerebral cortex of ischemic stroke patients were downloaded from the NCBI-GEO database (https://www.ncbi.nlm.nih.gov/geo/) with the accession ID GSE56267 (open to be acessed). Raw sequencing data in fastq format were processed with HISAT2, StringTie, and Ballgown [35]. The generated expression matrix was annotated using the annotation file downloaded from the BioMart tool of the Ensembl database [36]. Differential expression analysis was performed using the empirical Bayesian algorithm in the limma package [37] in R statistical software (https://www.r-project.org/). Significantly differentially expressed genes were defined as absolute values of log2-transformed fold change (logFC) > 1 and FDR P value < 0.05.

### Optimization of the C-T-P network with the MOO model

The C-T-P network was optimized using the MOO model. The purpose of optimization is to extract targets that are strongly correlated with compounds and pathogenic genes and are dysregulated in stroke patients. The following are some definitions: let $\varvec{C}$ be the set of filtered compounds, $c$ be a compound in $\varvec{C}$; $\varvec{T}$ be the set of all predicted targets, $t$ be a target in $\varvec{T}$; and $\varvec{P}$ be the set of all stroke pathogenic genes,$p$ be a pathogenic gene in $\varvec{P}$. Note that there are essential common proteins between $\varvec{T}$ and $\varvec{P}$. Let ${\varvec{T}}_{\varvec{c}}$ be the set of predicted targets for $c$, and let ${\varvec{P}}_{\varvec{t}}$ be the set of stroke pathogenic genes interacting with $t$. The following is the detailed optimization process:

Step 1. Let ${Score}_{{exp}_{t}}$ be the differential expression score for each target $t$. If the target is also a stroke pathogenic gene, ${Score}_{{exp}_{t}}=\left|\text{log}\ {FC}_{t}\right|$; if the target is not a stroke pathogenic gene, ${Score}_{{exp}_{t}}=\sum \left|\text{log}\ {FC}_{p}\right|/{n}_{t}$, where ${n}_{t}$ is the number of pathogenic genes interacting with $t$.

Step 2. Let ${Score}_{{cor}_{t}}$ be the correlation score with stroke pathogenic genes for each target $t$. If the target is also a stroke pathogenic gene, ${Score}_{{cor}_{t}}=1$; if the target is not a stroke pathogenic gene, ${Score}_{{cor}_{t}}=\sum \left|cor\left(t,p\right)\right|/{n}_{t}$, where $\left|cor\left(t,p\right)\right|$ indicates the absolute value of the correlation coefficient between target $t$ and pathogenic gene $p$, and ${n}_{t}$ is the number of pathogenic genes interacting with target $t$.

Step 3. Let ${Score}_{{deg}_{t}}$ be the degree score based on the network degree in the C-T-P network for each target $t$. Let ${N}_{CT}$ be the original compound–target network and ${N}_{TP}$ be the original gene-gene interaction network containing only targets and stroke pathogenic genes. Note that a target in ${N}_{CT}$ may not belong to ${N}_{TP}$. ${D}_{T}\left(\cdot\right)$ is a function to calculate the degree of the target in the network. ${M}_{T}\left(\cdot\right)$ is a function to calculate the maximum degree minus the minimum degree in the network. The calculation formula of ${Score}_{{deg}_{t}}$ is:

$${Score}_{{deg}_{t}}=\frac{{D}_{T}\left({N}_{{CT}_{t}}\right)-{\left[{D}_{T}\left({N}_{CT}\right)\right]}_{min}}{{M}_{T}\left({N}_{CT}\right)}\cdot\frac{{D}_{T}\left({N}_{{TP}_{t}}\right)-{\left[{D}_{T}\left({N}_{TP}\right)\right]}_{min}}{{M}_{T}\left({N}_{TP}\right)}$$

Step 4. Let ${Score}_{{path}_{t}}$ be the pathway score calculated based on the enriched pathways for each target $t$. Let ${P}_{T}\left(t\right)$ be a function to calculate the KEGG pathways in which target $t$ is involved, and let ${P}_{T}\left(\varvec{T}\right)$ be the union set of KEGG pathways in which all targets are involved. The calculation formula of ${S}_{{path}_{j}}$ is:

$${Score}_{{path}_{t}}=\frac{{P}_{T}\left(t\right)\cap {P}_{T}\left(\varvec{T}\right)}{{P}_{T}\left(t\right)\cup {P}_{T}\left(\varvec{T}\right)}$$

Step 5. Normalize (set the value range to 0 and 1) ${Score}_{exp}$, ${Score}_{cor}$, ${Score}_{deg}$, and ${Score}_{path}$ and obtain ${Score'}_{exp}$, ${Score'}_{cor}$, ${Score'}_{deg}$, and ${Score'}_{path}$. The MOO score is calculated as:

$${Score}_t={Score'}_{{exp}_t}+{Score'}_{{cor}_t}+{Score'}_{{deg}_t}+{Score'}_{{path}_t}$$

targets were sorted according to the ${Score}_{t}$ from high to low, the top 50% of targets were selected, and the C-T-P network was reconstructed. The optimized network was used for further analysis.

### GO and KEGG enrichment analysis

GO terms of biological process, cellular component, molecular function, and human gene information were downloaded from the QuickGO database (https://www.ebi.ac.uk/QuickGO/) [38]. The reference human genes and pathways were obtained from the Kyoto Encyclopedia of Genes and Genomes (KEGG) database (http://www.kegg.jp/) [39]. GO terms and KEGG pathways with fewer than 10 genes were removed. The enrichment analysis was performed using the hypergeometric test. An FDR-corrected P value ≤ 0.05 was considered significantly enriched.

### Comparison of the MOO method with other models

The degree model, closeness model, and betweenness model [40] were used to optimize the constructed C-T-P network and compared to the MOO model. The optimization performance of different models was compared from the following 5 aspects: (1) the coverage of Essential common proteins; (2) the coverage of the top 100 enriched KEGG pathways; (3) the coverage of the top 1000 enriched GO biological processes (BP); (4) the average regulating intensity; and (5) the cumulative differential expression. Genes in the optimized network in different models were used to perform KEGG pathway and GOBP enrichment analyses, and the reference KEGG pathways and GOBPs were enriched using ECPs. The average regulating intensity was calculated as the mean Pearson’s correlation coefficient for gene‒gene pairings in the optimized network. Cumulative differential expression is defined as the sum of the absolute values of logFC of Essential common proteins in the optimized network.

### Calculation of the cumulative target coverage score

We first calculated the target coverage score of a single compound. Let ${Score}_{c}$ be the target coverage score of compound $c$, ${T}_{c}$ be the set of targets interacting with compound $c$, and ${n}_{c}$ be the number of targets in ${T}_{c}$. The following is the calculation formula:

$${Score}_{c}=\sum _{t=1}^{{n}_{c}}{Score}_{t},t\in {T}_{c}$$

For the cumulative target coverage score of multiple compounds, we first built a list of combinations of different compounds and then calculated the coverage score of each combination using the above formula. The combination containing the same number of compounds with the strongest coverage score was defined as the optimal combination. Finally, we sorted these optimal combinations in ascending order of coverage score and selected the lowest compound combination whose cumulative target coverage score reached 90% of the coverage score of all compounds in the optimized network.

### Calculation of the normalized cumulative importance of enriched pathways

The normalized cumulative importance of the pathway is defined as the sum of the normalized MOO score of targets in the pathway. Let ${Importance}_{p}$ be the normalized cumulative importance of the pathway and $m$ be the number of targets in the pathway. The calculation formula is as follows:

$${Importance}_{p}=\frac{1}{m}\sum _{t=1}^{m}{Score}_{t}$$

All pathways are sorted according to the ${Importance}_{p}$, and the pathways with larger ${Importance}_{p}$ are considered to be the main pathways affected by THSWD.

### Molecular docking

The crystal structures of compound target proteins were downloaded from the Protein Data Bank (PDB) database (https://www.rcsb.org/) [41]. The SMILES molecular formula was converted into “.pdb” format by Discovery Studio version 2016. Proteins and compounds in “.pdb” format were converted to “.pdbqt” format for molecular docking using the OpenBabel Toolkit. The center position (x, y, z coordinates) and radius of potential binding sites of target proteins were calculated using the From Receptor Cavities tool in Discovery Studio. Molecular docking analysis of the filtered compounds and proteins was performed using AutoDock Vina 1.1.2 [42] with its default parameters, and the affinity (docking energy) less than − 6 kcal/mol of each docking pair was chosen as the docking result.

### Cell culture

The HT22 cell line (murine hippocampal cells) was obtained from CHI Scientific (Shanghai, China). The cells were maintained in a humidified atmosphere at 37 °C, 5% CO2, and complete culture medium (DMEM supplemented with 10% FBS).

### Oxygen–glucose deprivation and reoxygenation (OGD/R) modelin vitro.

Oxygen and glucose deprivation/reoxygenation (OGD/R) is a well-established model to mimic the ischemia/reperfusion conditions of cells in vitro [43]. Specifically, the cells were washed three times with PBS, digested by trypsin, and plated at 20,000 cells/200 µl medium per well of a 96-well plate. After 24 h, the cells were changed to low-glucose DMEM (1 g/l glucose) and incubated in a hypoxic environment at 1% O2, 5% CO2, and 37 °C for 18 h. After the OGD period, the cells were incubated in complete culture medium with compounds at concentrations of 0, 1.56, 3.12, 6.25, 12.5, 25, 50, and 100 µM under normoxic conditions for 24 h. In addition, different concentrations of compounds were dissolved in complete culture medium during the period of reoxygenation.

### Cell viability assay

Cell viability assays were carried out with a Cell Counting Kit-8 (RiboBio, China) following the methods described earlier [44]. In brief, 10 µl of CCK-8 solution in 100 µl of complete culture medium was added to each well of a 96-well plate and further incubated for 3 h. Then, the absorbance was measured at 450 nm optical density in a microplate reader (KC junior, BioTek, USA). The cell viability was calculated by the mean of the optical density values in 6 replicate wells.

### Materials

Fetal bovine serum (FBS) and Dulbecco’s modified Eagle’s medium (DMEM) were purchased from Thermo Fisher Biochemical Products (Beijing) Co., Ltd. Hypoxic bags were purchased from Mitsubishi Gas Chemical Company, Inc. (Japan). Cell Counting Kit-8 (CCK-8) was purchased from Dojindo Laboratories (Japan). All compounds were purchased from Jiangsu Yongjian Pharmaceutical Technology Co., Ltd. (Jiangsu, China), and the purity was higher than 95%.

### Statistical analysis

The same to the methods described in our earlier study [44], R statistical software version 4.0.3 was used for the statistical analysis of the experimental data. Outliers in each repeat higher than twofold of the standard deviation were eliminated. Independent sample t tests were used to compare the individual effects of different concentrations of drugs on cell viability. One-way ANOVA and LSD post hoc tests were used to test the synergistic effects of the active drugs on cell viability. A P value less than 0.05 was considered statistically significant.

## Results

### Original and filtered potential active compounds in THSWD

**Fig. 2:**
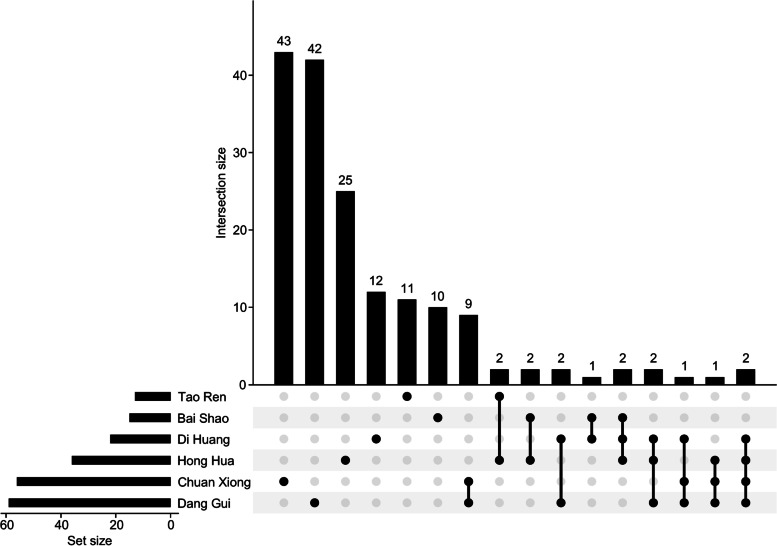
Compounds after ADMET screening in herbs in THSWD. Lines between dots indicate the compounds’ intersection of botanical drugs

A total of 1294 compounds among 6 herbs (*Angelica sinensis*, *Ligusticum chuanxiong*, *Carthamus tinctorius*, *Rehmannia glutinosa*, *Paeonia lactiflora*, and *Prunus persica*) were collected in this study. The pharmacological properties of these compounds were predicted (Table S1). According to our screening criteria, 295 potentially active compounds among these 6 herbs were retained. The numbers of screened potential active compounds in *Angelica sinensis*, *Ligusticum chuanxiong*, *Carthamus tinctorius*, *Rehmannia glutinosa*, *Paeonia lactiflora*, and *Prunus persica* were 43, 42, 25, 12, 11, and 10, respectively (Fig. 2, Table S2). There were only 9 compounds shared by Angelica sinensis and Ligusticum chuanxiong, and 2 compounds were shared by Angelica sinensis, Ligusticum chuanxiong, *Carthamus tinctorius* and *Rehmannia glutinosa*.

### C-T-P network construction and optimization

**Fig. 3:**
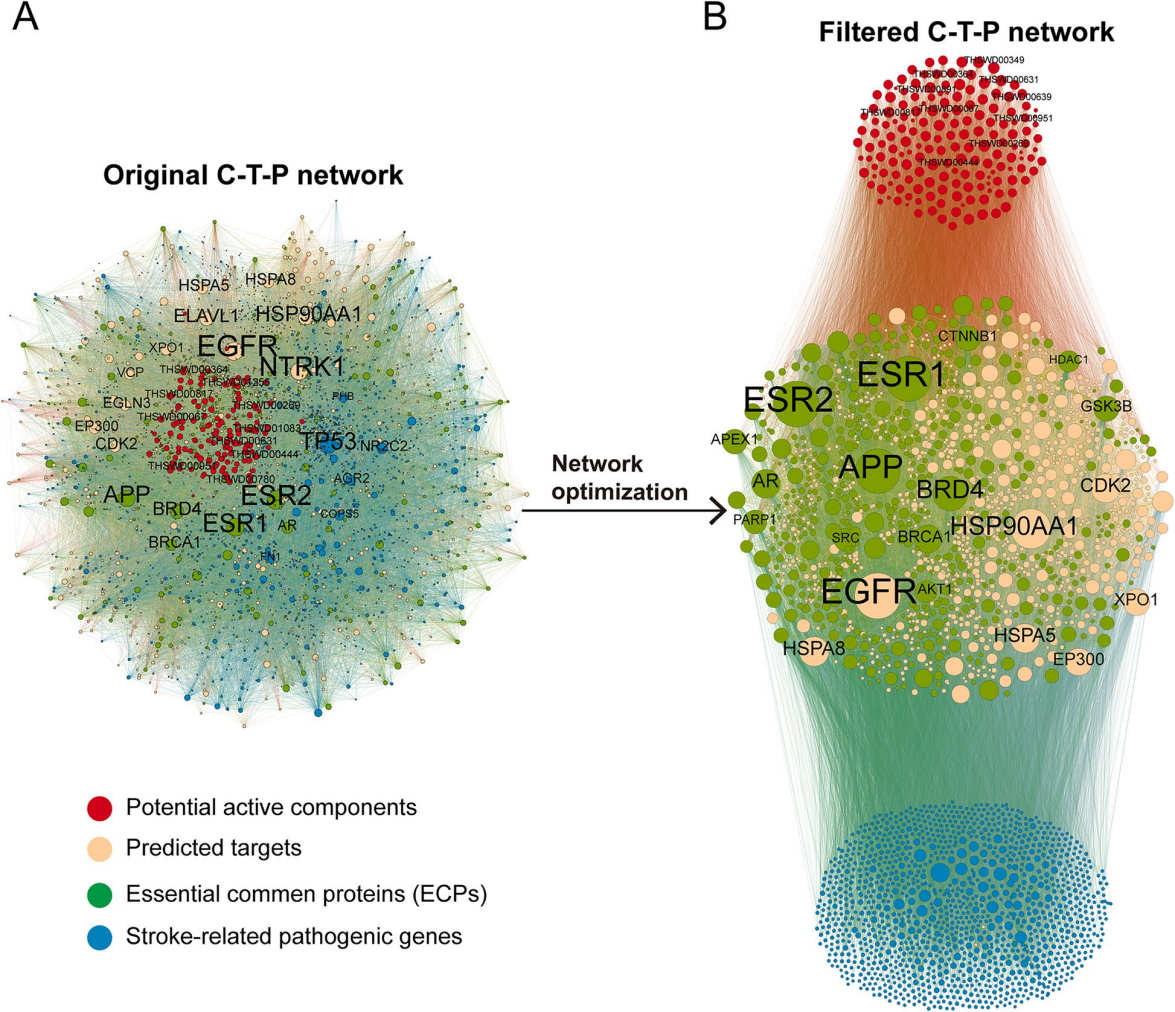
The original and optimized C-T-P network. The red, yellow, blue, and green points indicate potential active compounds, predicted targets, stroke pathogenic genes, and ECPs, respectively. Compounds and genes with a high degree of expression were highlighted and labeled

We first removed the duplicated compounds and then performed target prediction. SEA, HitPickV2, and SwissTargetPrediction were used to predict the targets of the remaining 167 compounds. The prediction results showed that these compounds may act on 10 to 160 potential targets, and the number of unique predicted targets was 1627 (Table S3). A total of 2143 unique stroke pathological genes reported in ischemia were collected (Table S4). These targets and pathological genes were mapped with the BioGRID database to obtain protein-protein interaction information. Then, the compound–target network and protein-protein interaction network were combined to construct the C-T-P network. The original C-T-P network contained 17,126 compound–target pairs and 44,878 protein-protein interactions (Fig. 3A). In the original C-T-P network, 534 proteins were both target proteins and pathogenic genes, which were defined as ECPs. KEGG pathway and GOBP enrichment analysis showed that these genes are mainly involved in neurodegeneration pathways and the regulation of various nervous system-related signaling pathways (Table S5-S6). Then, network optimization and active compound screening were used in the MOO method. The optimized C-T-P network contains 9,811 compound–target pairs and 29,199 protein-protein interactions, which are composed of 167 compounds, 1,467 predicted targets, and 1,758 stroke pathological genes (Fig. 3B). All the ECPs were retained in the optimized C-T-P network. The top 20 genes with the highest degree in the original and optimized networks are labeled. Among these top genes, there were 6 ECPs (AR, APP, BRCA1, BRD4, ESR1, and ESR2) also in the original network and 13 ECPs (AR, AKT1, APEX1, APP, BRCA1, BRD4, CTNNB1, ESR1, ESR2, HDAC1, GSK3B, PARP1, and SRC) in the optimized network. The results showed that the MOO model retained the vast majority of ECPs while removing unimportant targets.

### Comparison of the optimization performance between the MOO model and other models

**Fig. 4:**
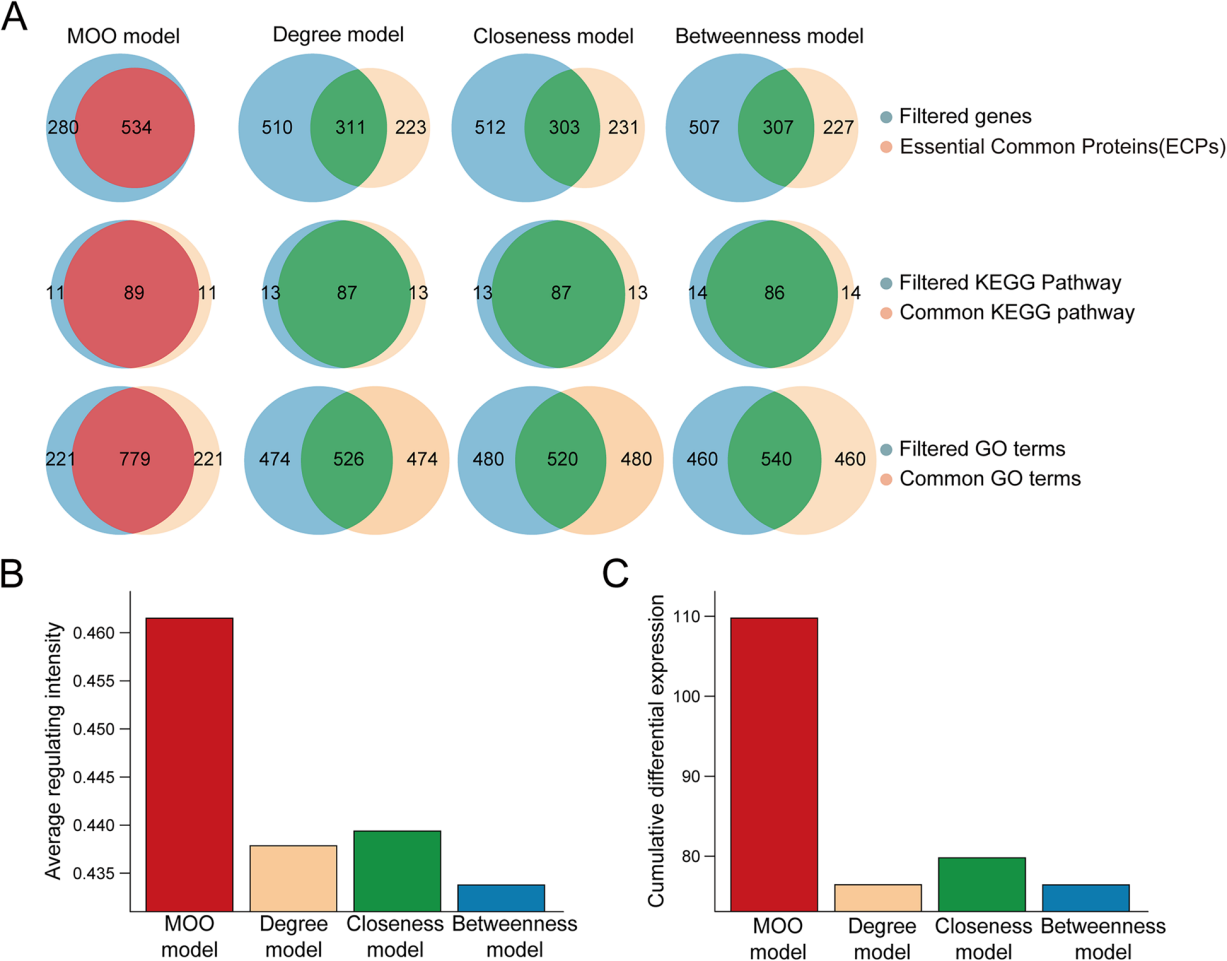
Optimization performance comparison of the MOO model with other models. (A) The overlap between filtered genes in the optimized C-T-P networks and ECPs, as well as their enriched KEGG pathways and GOBPs. (B) The average regulating intensity of the MOO and the other three models. (C) Cumulative differential expression of the MOO and the other three models

Unique genes in the optimized network by the MOO model, degree model, closeness model, and betweenness model were also subjected to ECP overlap, KEGG pathway, and GOBP enrichment analysis. The top 100 enriched KEGG pathways and the top 1000 enriched GOBPs were used to compare the model performance. The numbers of overlapping ECPs were 534, 311, 303, and 307 in the MOO model, degree model, closeness model, and betweenness model, respectively. The number of overlapping top 100 enriched KEGG pathways was 89, 87, 87, and 86 in these 4 models, respectively. The numbers of overlapping top 1000 enriched GOBPs were 779, 526, 520, and 540 in these 4 models, respectively. Furthermore, the values of the average regulating intensity and cumulative differential expression in the MOO model were higher than those of the other three models (Fig. 4). These results suggested that the optimization performance of the MOO model is better than that of the other models.

### Key functional component screening based on the cumulative target coverage score

**Fig. 5:**
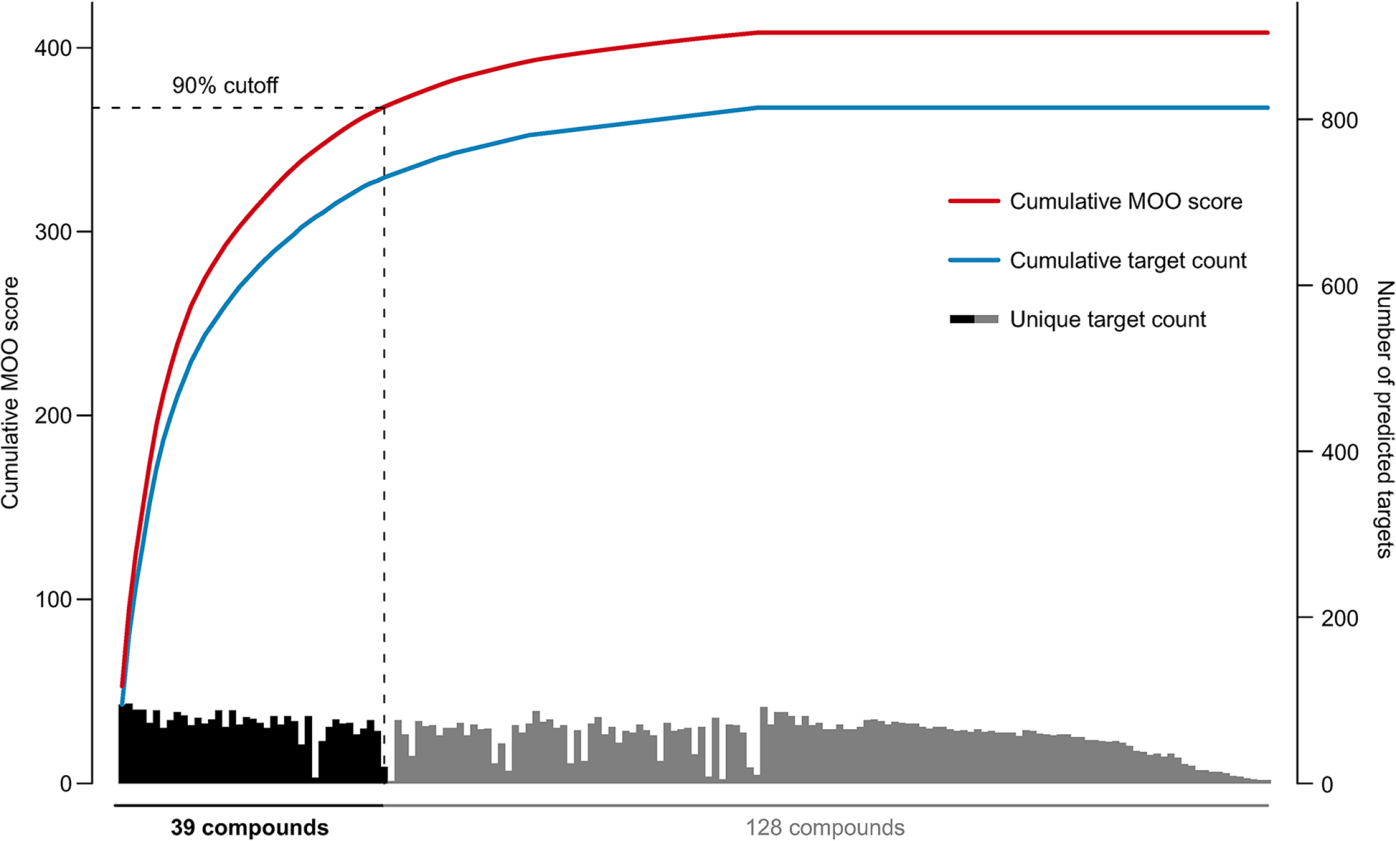
The cumulative target coverage scores and predicted target count of all potential active compounds in the optimized C-T-P network. A dashed horizontal line indicates the 90% cumulative target coverage score of all potential active compounds

**Fig. 6:**
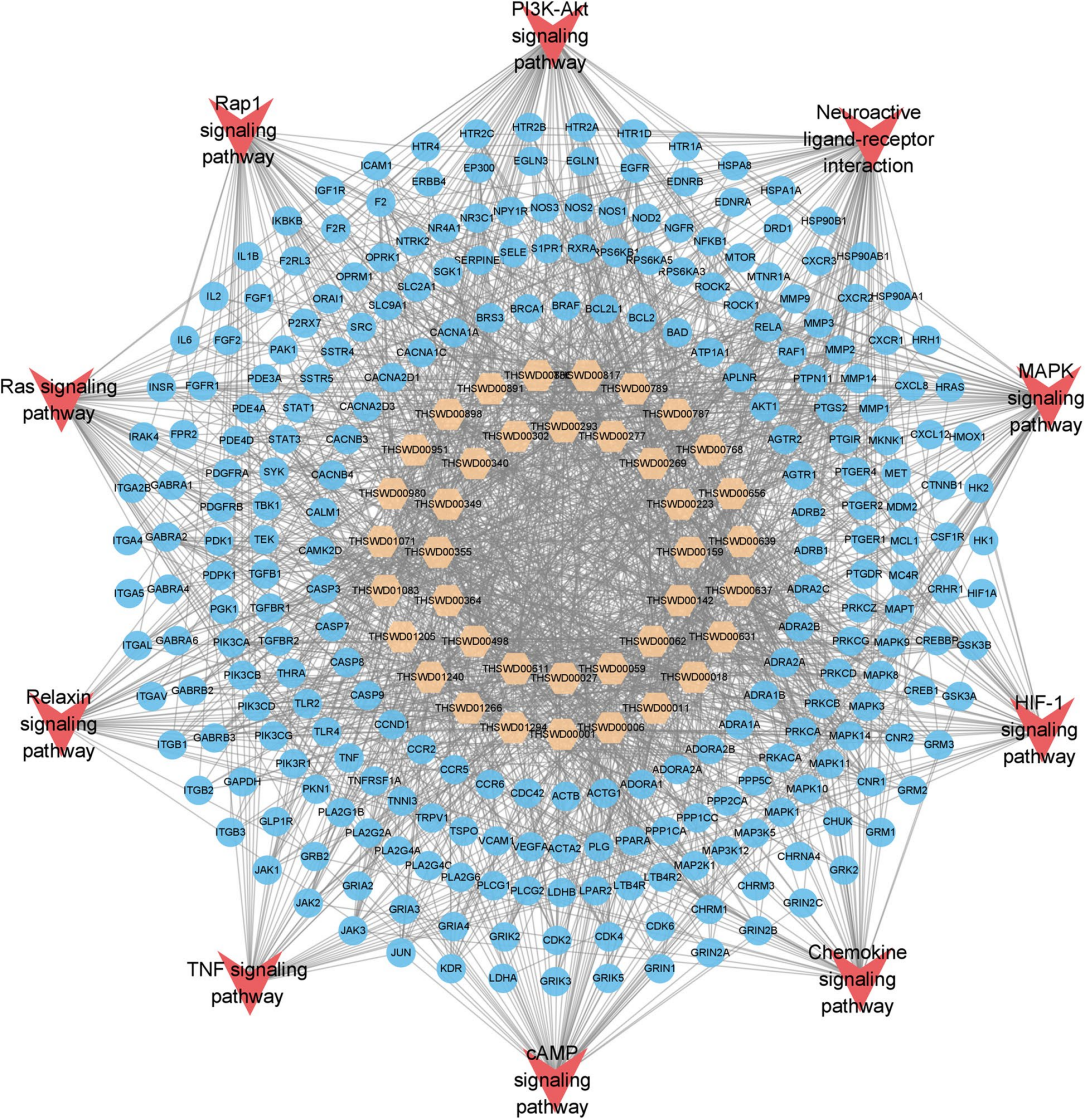
The compound-target-pathway network of the predicted targets and pathways of the final screened potential active compounds. The yellow shape represents the compounds, the blue shape represents the predicted targets, and the red shape represents the top 10 pathways affected by THSWD

We calculated the cumulative target coverage score of each compound and its combinations (Table S7). The cumulative target coverage scores of the combination of 39 compounds reached 90% coverage of all potential active compounds (Fig. 5; Table 2). Among these compounds, 33 compounds were derived from unique botanical drugs, and the other 6 compounds were derived from 2 or more herbs. Then, we calculated the normalized cumulative importance of enriched pathways based on the normalized cumulative importance (Table S8). The final screened 39 key functional components, predicted targets, and the related top 10 pathways are shown with a compound-target-pathway network (Fig. 6). Multiple stroke-related pathways were affected by these compounds, such as the PI3K-Akt signaling pathway, neuroactive ligand-receptor interaction, cAMP signaling pathway, Rap1 signaling pathway, and MAPK signaling pathway.

**Table 2:** Information of final screened 39 key functional compounds in THSWD.

ID	Name	Formula	Herbs
THSWD00631	(3 S)-3-butyl-4,5,6,7-tetrahydro-3 H-2-benzofuran-1-one	C_12_H_18_O_2_	Chuan Xiong
THSWD00269	Senkyunolide B	C_12_H_12_O_3_	Dang Gui, Chuan Xiong
THSWD00951	Oroxylin A	C_16_H_12_O_5_	Tao Ren
THSWD00891	3β,23-dihydroxy-olea-11,13(18)-dien-28-oic acid	C_30_H_46_O_4_	Bai Shao
THSWD00142	L-Proline	C_5_H_9_NO_2_	Dang Gui
THSWD00639	(3R)-3-butyl-4-hydroxy-3 H-2-benzofuran-1-one	C_12_H_14_O_3_	Chuan Xiong
THSWD00789	Rehmaglutin A	C_9_H_14_O_5_	Di Huang
THSWD00223	Vitamin A	C_20_H_30_O	Dang Gui, Chuan Xiong
THSWD00349	Chrysophanol	C_15_H_10_O_4_	Chuan Xiong
THSWD00787	Rehmapicroside	C_16_H_26_O_8_	Di Huang
THSWD00293	L-Methionine	C_5_H_11_NO_2_S	Dang Gui
THSWD00001	Decanoic acid	C_10_H_20_O_2_	Dang Gui, Chuan Xiong, Di Huang, Bai Shao, Hong Hua
THSWD00898	(Z)-(1 S,5R)-beta-pinen-10-yl-beta-vicianoside_qt	C_10_H_16_O	Bai Shao
THSWD01083	Matairesinol	C_20_H_22_O_6_	Hong Hua
THSWD00364	(3 S,4R)-3-butyl-4-hydroxy-4,5-dihydro-3 H-2-benzofuran-1-one	C_12_H_16_O_3_	Chuan Xiong
THSWD00011	Calycosin	C_16_H_12_O_5_	Dang Gui
THSWD00817	PyrethrinIi	C_22_H_28_O_5_	Bai Shao, Hong Hua
THSWD00980	D-Mandelic acid	C_8_H_8_O_3_	Tao Ren
THSWD00018	Bergapten	C_12_H_8_O_4_	Dang Gui
THSWD01240	Roseoside	C_19_H_30_O_8_	Hong Hua
THSWD00498	L-Bornyl acetate	C_12_H_20_O_2_	Chuan Xiong
THSWD00656	L-valyl-L-valinc-achydride	C_11_H_22_N_2_O_2_	Chuan Xiong
THSWD00611	Sinapinic acid	C_11_H_12_O_5_	Chuan Xiong
THSWD00886	(+)-trans-Myrtanol	C_10_H_18_O	Bai Shao
THSWD00027	Nodakenetin	C_14_H_14_O_4_	Dang Gui
THSWD00355	(1aS,4Z,6bR)-4-butylidene-1a,2,3,6b-tetrahydrooxireno[2,3-g] [2]benzofuran-6-one	C_12_H_14_O_3_	Chuan Xiong
THSWD00006	Citric acid	C_6_H_8_O_7_	Dang Gui
THSWD00768	Jioglutolide	C_9_H_14_O_4_	Di Huang
THSWD00159	Glycine	C_2_H_5_NO_2_	Dang Gui
THSWD00277	Dictyopterene C	C_11_H_18_	Dang Gui, Chuan Xiong
THSWD01294	4,6-Decadiyn-1-ol isovalerate	C_15_H_22_O_2_	Hong Hua
THSWD01205	ethyl (1 S,2R,3 S)-6,7-dimethoxy-3-methyl-4-oxo-1-(3,4,5-trimethoxyphenyl)-2,3-dihydro-1 H-naphthalene-2-carboxylate	C_25_H_30_O_8_	Hong Hua
THSWD01266	7,8-dimethyl-1 H-pyrazino[2,3-g]quinazoline-2,4-dione	C_12_H_10_N_4_O_2_	Hong Hua
THSWD00340	Butylphthalide	C_12_H_14_O_2_	Dang Gui, Chuan Xiong
THSWD01071	Syringin	C_17_H_24_O_9_	Hong Hua
THSWD00059	(1R,3 S)-Camphoric acid	C_10_H_14_O_4_	Dang Gui
THSWD00062	2,4-Dihydroxyacetophenone	C_8_H_8_O_3_	Dang Gui
THSWD00637	4-Iodoindoline	C_8_H_8_IN	Chuan Xiong
THSWD00302	[(2R)-2-formyloxy-3-phosphonooxypropyl] formate	C_5_H_9_O_8_P	Dang Gui

### Experimental verification of the key functional compounds

**Fig. 7:**
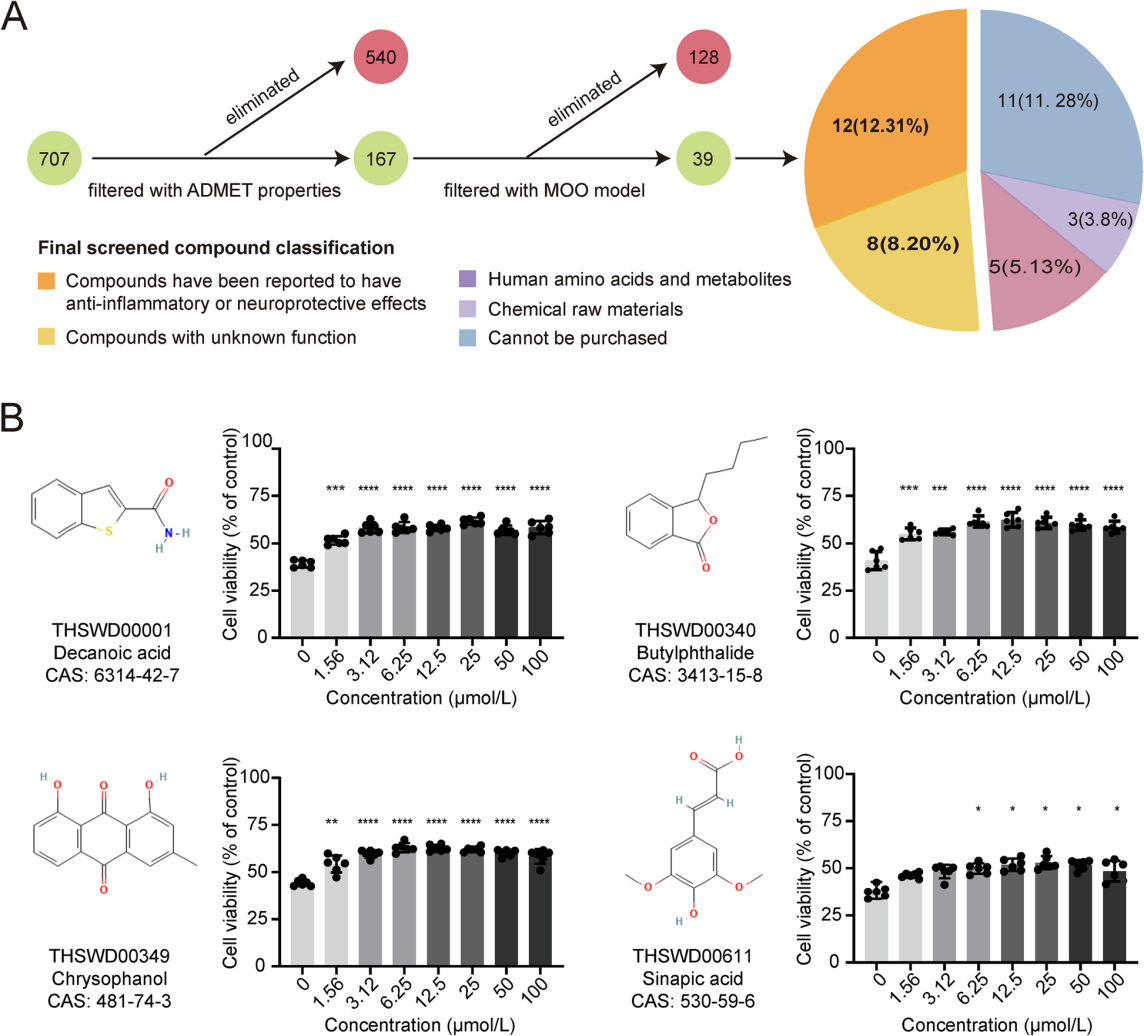
Experimental verification of the screened potential therapeutic compounds. (A) The screening process for compounds used for experimental validation. (B) Effects of decanoic acid, butylphthalide, chrysophanol, and sinapic acid on cell viability. The structure of the compound is shown on the left side of the bar plot. Significance: *P < 0.05, **P < 0.01, ***P < 0.001

The final screened 39 key functional compounds included 12 compounds that have been reported to have anti-inflammatory and neuroprotective effects, 8 compounds with unknown function, 5 compounds that belong to human amino acids and metabolites, 3 compounds known as chemical raw materials, and 11 compounds not available for purchase (Fig. 7A, Table S9). We used the in vitro OGD/R model to verify the 12 previously reported potential active compounds and 8 unreported compounds. The results showed that THSWD00001 (decoranoic acid), THSWD00340 (butylphthalide), THSWD00349 (chrysophanol), and THSWD00611 (sinapic acid) significantly increased cell viability from low to high concentrations compared to the control group after OGD/R (Fig. 7B). However, the other 16 compounds showed no significant effect on cell viability. In more detail, among the 20 compounds that were calculated and filtered, 4 compounds were tested to be effective. In fact, the efficiency of 20% is much higher than traditional drug screening methods. In the in vitro study, we only selected the most commonly used OGD/R model to simulate neuronal injury of hypoxia-reperfusion. Adding other neuronal injury models, such as H2O2 nerve injury model and glutamate nerve injury model, may improve the experimental efficacy of the compounds. The safety evaluation with normal HT22 cells and without OGD/R process showed that decanoic acid, butylphthalide, chrysophanol, and sinapic acid didn’t affect cell viability at concentrations less than 100 µmol/L (Supplementary Fig. 1).

### The binding mode of the effective compounds proven by experiments and their targets

**Fig. 8:**
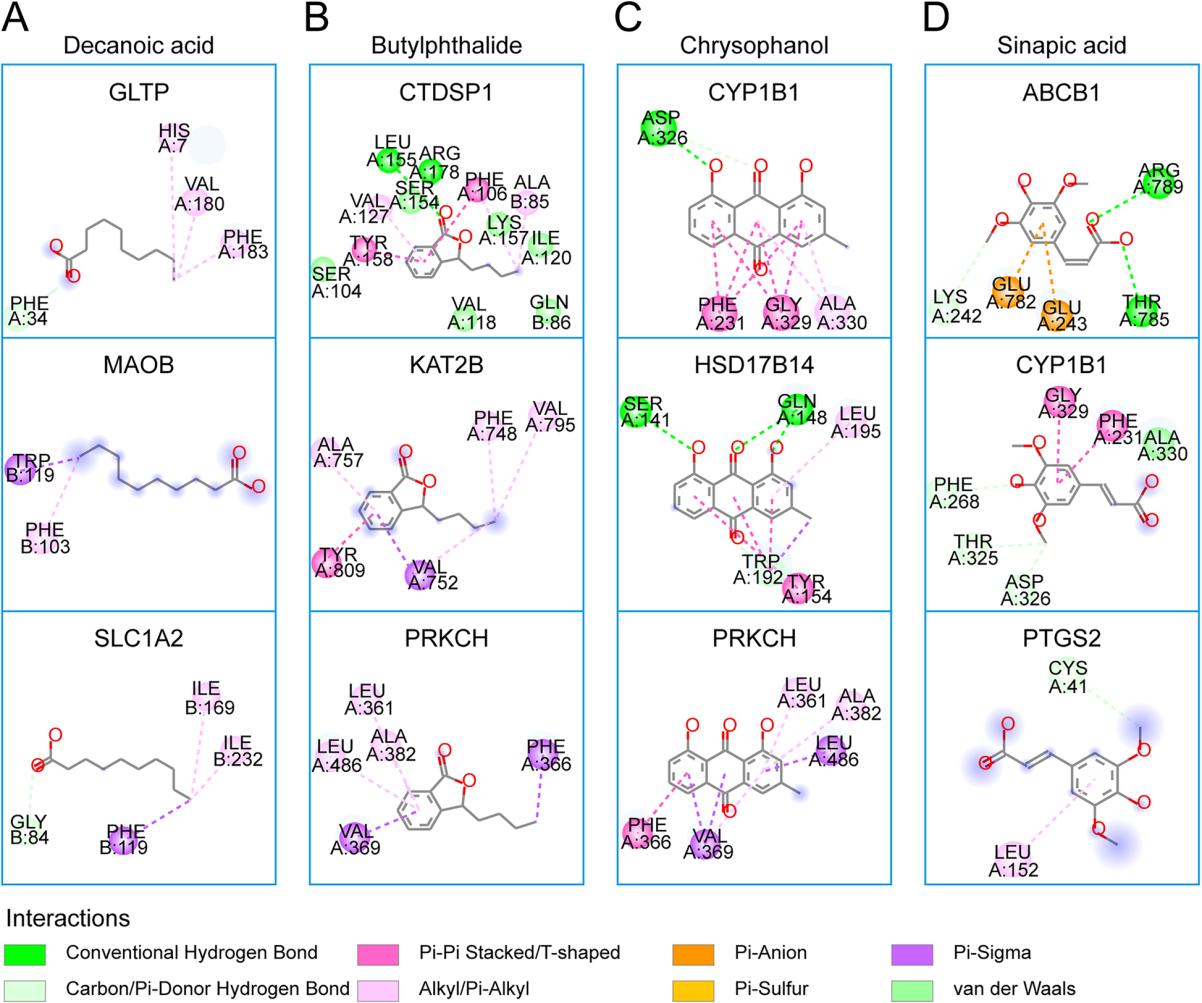
The 2D diagram of the interactions of four validated compounds (decoranoic acid, butylphthalide, chrysophanol, and sinapic acid) and their top 3 target proteins. The bonds between the compounds and the amino acid residues are indicated by colored dashed lines

The docking affinities of the above four compounds and their target proteins are shown in Table S10. The results showed that THSWD00001 (decanoic acid) may bind to 3 targets (MAOB, GLTP, and SLC1A2), THSWD00340 (butylphthalide) may bind to 5 targets (BCL2A1, CTDSP1, FOLH1, KAT2B, and PRKCH), THSWD00349 (chrysophanol) may bind to 15 targets (ALPL, BCL2, BCL2A1, CA2, CA5B, CTDSP1, CYP1B1, HSD17B14, JUN, LDHA, LDHB, MCL1, MIF, NR2F2, and PRKCH), and THSWD00611 (sinapic acid) may bind to 5 targets (ABCB1, CYP1B1, LY96, MIF, and PTGS2). The results showed that THSWD00349 (chrysophanol) has the most binding targets. In addition, the binding mode of each compound to the top 3 targets with the highest docking affinity is shown in Fig. 8. For example, the amino acid residues of GLTP that interact with decanoic acid are His7, Phe34, Val180, and Phe183; the amino acid residues of CTDSP1 that interact with butylphthalide are Ala85, Gln86, Ser104, Phe106, Val118, Ile120, Val127, Ser154, Leu155, Lys157, Tyr158, and Arg178; the amino acid residues of CYP1B1 that interact with chrysophanol are Phe231, Asp326, Gly329, and Ala330; and the amino acid residues of ABCB1 that interact with sinapic acid are Lys242, Glu243, Glu782, Thr785, and Arg789.

## Discussion

TCM has a long history and rich experience in the treatment of complex diseases such as stroke, which provides new directions for us to screen effective components for nerve protection after ischemic stroke [45–47]. According to the theory of TCM, a formula for treating stroke usually composed of multiple botanical drugs, with thousands of compounds. Thus, it is important to develop accurate and inexpensive computational analysis methods to screen the key functional ingredients for further experimental verification and mechanism exploration [46–48].

At present, many formulas have been used in clinical treatment, and their mechanisms of action is also being explored, among which THSWD has attracted our attention. First of all, a lot of previous clinical studies in China have shown that THSWD has a good effect on the rehabilitation of patients after stroke. For example, THSWD combined with Western medicine was reported a therapeutic effect on ischemic patients with nerve defect and blood stasis syndrome [49]. In the early treatment of acute cerebral infarction, the combination of THSWD and Ditan decoction was reported to increase blood flow and reduce the level of inflammatory factors in the serum of patients [50]. Moreover, in the recovery stage, THSWD could significantly improve the daily life ability and neurological function of patients [51]. In addition, several previous pharmacological studies have also confirmed that THSWD could exhibit potential neuroprotective effects and have therapeutic potential both in vitro and in vivo. Wang reported that THSWD could significantly improve cerebral infarction and promote angiogenesis in the ischemic area of rat model, and the mechanism was related to the regulation of ET-1, Ang-1, and VEGF content in the serum of rats with cerebral ischemia [52]. In addition, Zhang reported that THSWD had a significant effect on rescuing nerve function in rats through cerebral ischemia and reperfusion injury, and mechanistic exploration showed that THSWD could promote angiogenesis in cerebral ischemia sites via the PI3K/AKT signaling pathway [53].

In this study, we propose a network analysis strategy for screening key functional compounds of THSWD in the treatment of ischemic stroke. This strategy includes optimizing the targets of complex C-T-P network through MOO model, and then collecting compounds with 90% target coverage by accumulating target coverage scores. Our approach has two advantages: (1) our proposed method comprehensively considered multiple features of target genes, including transcriptome changes, associations with pathogenic genes, network properties, and enriched pathways. Compared with single-feature optimization, the key target genes screened by the MOO model are more representative. (2) The model we proposed has broad applicability; it does not depend on a specific prescription, disease, or gene expression signature and can be applied to the screening of active compounds in other TCM prescriptions.

Based on the new model proposed and used in this study, we filtered 39 key functional compounds. Among them, 12 compounds have been reported to have anti-inflammatory and neuroprotective effects, which are closely related to ischemic injury of the brain. A high percentage (12/39) of compounds with potential effects for stroke treatment validated the reliability of the MOO model once again (Fig. 7A). Furthermore, 20 compounds (12 compounds with reported anti-inflammatory or neuroprotective effects and 8 compounds with unknown functions) were tested with the OGD/R model in the hope of finding potential neuroprotective drugs for ischemic stroke through further experiments. The results revealed that 4 compounds, decanoic acid, butylphthalide, chrysophanol, and sinapic acid, could promote nerve cell survival in the OGD/R process. Of the 4 compounds, decanoic acid and sinapic acid were first found to have a potential therapeutic effect on ischemic stroke. Other compounds, including butylphthalid and chrysophanol, have been reported in previous literature. The experimental results showed that the MOO model indeed could reduce the experimental scope and improve the success rate of screening potential therapeutic drugs from the TCM formula compared with traditional methods.

According to previous literature, butylphthalide is a compound isolated from the seeds of celery Apium graveolens Linn [54]. To date, dl-3-N-butylphthalide, a synthetic variation of l-3-N-butylphthalide, remains the only clinically approved anti-ischemic agent in China [55]. Extensive studies have shown that butylphthalid exerts multitarget effects on stroke through a variety of mechanisms, including oxidative stress, mitochondrial dysfunction, apoptosis, and inflammation [54–56]. In addition, chrysophanol was reported to promote neurological recovery by downregulating the expression of IL1 and IL6 to limit microglia-mediated neuroinflammation and inhibit ROS production after ischemic stroke in mice [57–59].

Unlike butylphthalid and chrysophanol, decanoic acid and sinapic acid both have not been reported in stroke-related studies. However, according several researches reported, decanoic acid and sinapic acid still were known the role as anti-inflammatory, antioxidant and neuroprotective agent in some neuropathies. Decanoic acid was a C10 straight-chain saturated fatty acid, which was reported to promote GABA synthesis in neurons via elevated glutamine supply, reduce oxidative stress levels in neuroblastoma cells, and control seizure through direct AMPA receptor inhibition [60–62]. Sinapic acid is a natural herbal compound containing phenolic acid, which was found attenuating KA-induced hippocampus cell death through its GABA receptor activation, decreasing oxidative stress to save neurons of hemi-parkinsonian rat, and alleviating neuroinflammatory effects by inhibiting the increase in COX-2 and IL-1β [63–65].

## Conclusions

In conclusion, this study provides a methodological reference for the screening of potential therapeutic compounds of TCM, and we screened several active compounds in THSWD for the treatment of stroke. The neuroprotective effect of decanoic acid and sinapic acid was first found in this study and preliminarily verified with an OGD/R experiment in vitro. Admittedly, there are some limitations to this study. First, the compounds in botanical drugs we collected in the public TCM databases may not be sufficient; second, the active effect of compounds was only validated in vitro, without animal experiments; finally, we did not consider the metabolic changes of these compounds in the human body.

## Supplementary Information

**Additional file 1.**

**Additional file 2.**

## Data Availability

The datasets used and analysed during the current study are available from the corresponding author on reasonable request. And most data generated or analysed during this study are included in this published article and its supplementary information files. publicly available data are from the Traditional Chinese Medicine Systematic Pharmacology (TCMSP, https://tcmsp-e.com/) database;; the Traditional Chinese Medicine Information Database (TCMID, http://bidd.group/TCMID/); and the Traditional Chinese Medicine and Active Ingredient Database (TCMAID, http://www.organchem.csdb.cn/scdb/main/tcm_introduce.asp); DisGeNet database (https://www.disgenet.org/); NCBI-GEO database (https://www.ncbi.nlm.nih.gov/geo/) with the accession ID GSE56267.

## Abbreviations

TCM: Traditional Chinese medicine
C- T-P: D- compound–target–pathogenic gene
E- MOO: F- multiobjective optimization
G- OGD/R: H- oxygen-glucose deprivation and reoxygenation
I- ECPs: J- essential common proteins
