# Supplementary material for: Screening of key functional components of Taohong Siwu Decoction on ischemic stroke treatment based on multiobjective optimization approach and experimental validation
Source: BMC Complement Med Ther. 2023 Jun 1;23:178. doi: 10.1186/s12906-023-03990-1 (PMC10234048; doi:10.1186/s12906-023-03990-1)
Supplement: Supplementary file 2 — Additional file 2. [file 12906_2023_3990_MOESM2_ESM.pdf]

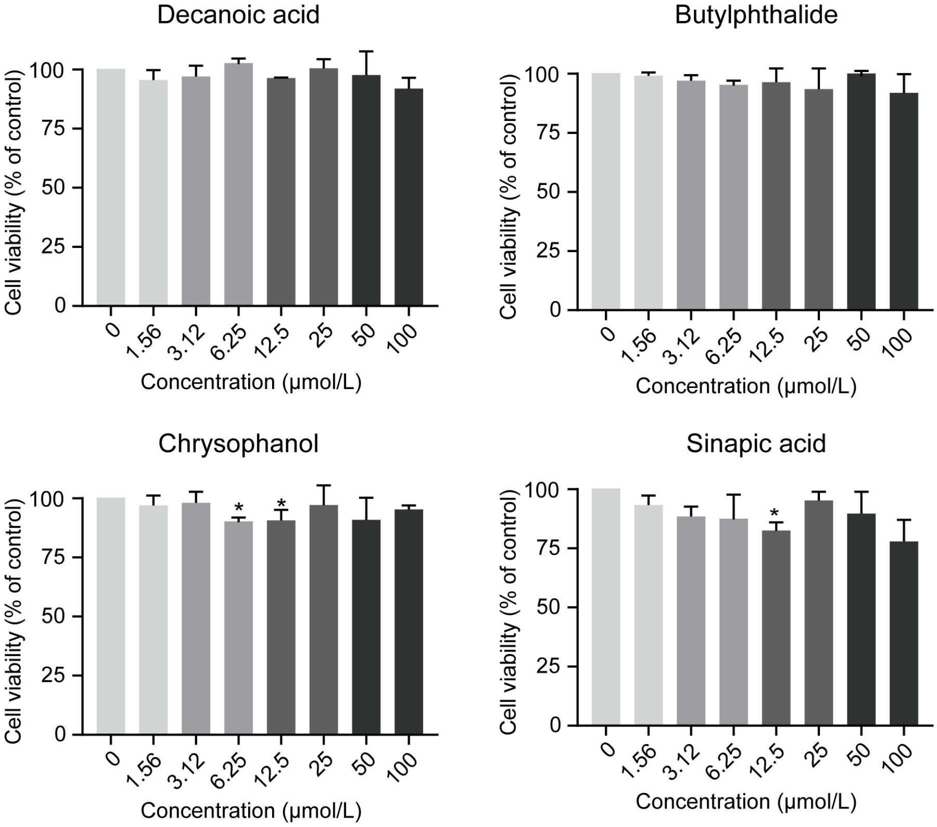

Supplementary figure1. The safety evaluation of decanoic acid, butylphthalide, chrysophanol, and sinapic acid with normal HT22 cells and without the OGD/R process. Data were reported as mean±SD (n=3). The cells with 0 concentration were as control group. Significance: *P < 0.05, **P < 0.01, ***P < 0.001.
